# Establishment of a human cell line with a surface display system for screening and optimizing Na^+^-taurocholate cotransporting polypeptide-binding peptides

**DOI:** 10.3389/fmicb.2022.920280

**Published:** 2022-08-17

**Authors:** Pei-yun Wang, Xue Yang, Lin Guo, Yu-wei Wang, Wen-lu Zhang, Yu-xue Sun, Jie Li, Chun-yang Gan, Shao-yuan Long, Jia-jun Liu, Shu-ying Fan, Ai-long Huang, Jie-Li Hu

**Affiliations:** ^1^Key Laboratory of Molecular Biology on Infectious Diseases, Ministry of Education, Chongqing Medical University, Chongqing, China; ^2^Department of Laboratory Medicine, Chongqing Hospital of Traditional Chinese Medicine, Chongqing, China; ^3^Laboratory for Diagnosis and Treatment of Infectious Diseases Integrated Traditional Chinese and Western Medicine, Chongqing Hospital of Traditional Chinese Medicine, Chongqing, China

**Keywords:** mammalian cell surface display, FACS, peptides, NTCP, screening and optimization

## Abstract

One of the most desirable targets for HBV medications is the sodium taurocholate cotransporting polypeptide (NTCP), an entry receptor for the hepatitis B virus (HBV). N-myristoylated preS1 2–48 (Myrcludex B or Hepcludex), an NTCP-binding peptide from the large surface protein of HBV, has been developed as the first-in-class entry inhibitor. However, its relatively large molecular weight contributes to increased immunogenicity and antibody production. As a result, it is preferable to look for an NTCP-binding peptide with a smaller size. To do this, we developed a human cell surface display strategy and screened peptides based on preS1-21. PreS1-21 (genotype D) was extended by 7 random amino acids and fused with mCherry and FasL transmembrane domain. The pooled constructs were transfected into HEK293 cells by using the transposon/transposase system to create a library displaying various peptides on the cell surface with red fluorescence. On the other hand, we expressed NTCP protein fused with EGFP on HEK293 and used the membrane lysate containing NTCP-GFP as the bait protein to select peptides with increased NTCP affinity. After 7 cycles of selection, the deep sequencing results revealed that some polypeptides were more than 1,000 times enriched. Further screening of the mostly enriched 10 peptides yields the peptide preS1-21-pep3. Replacing the preS1-21 sequence of preS1-21-pep3 with those from different genotypes demonstrated that the consensus sequence of genotype A–F had the best performance. The peptide (Myr-preS1-21-pep3) was synthesized and tested on the HepG2-NTCP cell model. The results showed that Myr-preS1-21-pep3 is approximately 10 times more potent than the initial peptide Myr-preS1-21 in preventing HBV infection. In conclusion, we developed a new strategy for screening peptides binding to membrane proteins and identified a new NTCP-binding peptide with a much smaller size than Hepcludex.

## Introduction

Chronic hepatitis B virus (HBV) infection is a public health problem worldwide. Although nucleotide analogs that inhibit viral reverse transcriptase are used clinically as anti-HBV agents, the emergence of drug-resistant viruses necessitates the development of new anti-HBV agents interfering with other targets (Kim et al., 2010). With the discovery of the Na^+^-taurocholate cotransporting polypeptide (NTCP) (Yan et al., 2012) as the HBV receptor, strategies have been developed to prevent HBV entry. Among these entry inhibitors targeting NTCP, N-myristoylated preS1 2–48 (commercial name Hepcludex), which integrates 2–48 amino acids of the N-terminal preS1 of all HBV genotypes based on type D, has a significant therapeutic effect (Schulze et al., 2010; Passioura et al., 2018). Hepcludex has the disadvantage of having a relatively large molecular weight, contributing to increased immunogenicity and antibody production (Passioura et al., 2018). Consequently, it is preferable to search for a smaller NTCP-binding peptide.

Surface display techniques, such as phage display, yeast cell surface display, and ribosome display, are frequently used to screen peptides that bind to specific proteins (Park, 2020). However, screening peptides that interact with NTCP may be problematic using standard surface display techniques. First, these peptides must be myristoylated to function. Proteins expressed from *E. coli* would lack posttranslational modifications like N-glycosylation and N-myristoylation (Cummings, 2009; Park, 2020). Glycosylation or myristoylated modifications may be produced in yeast cells, but the patterns are different from those found in mammalian cells (Lee et al., 2013; Wang et al., 2021). On the other hand, as a membrane protein, it is difficult for NTCP to be expressed and purified as intact membrane proteins from prokaryotic cells. In addition, once removed from the membrane, membrane proteins may be unable to reassemble into their normal structure, rendering them unsuitable as screening targets.

To tackle these challenges, surface display strategies based on human cells have been developed recently (Crook et al., 2017; Chan et al., 2020; Ivanusic et al., 2020). Here, we set up a system suitable for the screening of NTCP-binding peptides. We first demonstrated the feasibility of the system by using the FRB-FKBP12 interaction system. Next, we screened peptides with improved affinity for NTCP based on preS1-21. Seven cycles of selection yield the 27-amino acid peptide preS1-21-pep3, which is approximately 10 times more potent than the initial peptide preS1-21.

## Materials and methods

### Plasmids

Plasmid GFP–FRB expresses an FRB (FKBP12-rapamycin binding) and GFP fusion protein. Plasmid mCherry-FasL-FKBP12 expresses FKBP12 (FK506-binding protein) fused with mCherry and the FasL (Fas ligand) transmembrane domain (Crook et al., 2017). Plasmid NTCP–GFP expresses NTCP fused with GFP at the C-terminus. Plasmid NTCP d157-165-GFP was constructed based on NTCP-GFP by deleting the sequence between 157th aa and 165th aa of NTCP that has been proven to be essential for preS1 binding and viral infections (Yan et al., 2012). These plasmids were constructed based on the backbone of pCH9 (Nassal, 1992) by using the Golden gate cloning method (Engler et al., 2008, 2009). LentiCRISPRv2, psPAX2, and Pmd2.0G were constructed by Feng Zhang Lab (Sanjana et al., 2014). Plasmid Lenti-NTCP-GFP-blast is a lentivirus construct based on the backbone of LentiCRISPRv2 for the preparation of the lentivirus that expresses NTCP-GFP and the blasticidin-resistant gene. Plasmid Lenti-NTCP-Cherry-blast is a lentivirus vector to prepare the lentivirus that expresses NTCP–mCherry. Plasmid NTCP 267S/F-mCherry expresses NTCP-mCherry with an amino acid mutation at the 267th aa of NTCP. NTCP d157-165-mCherry expresses NTCP–mCherry with the deletion of aa 157 to aa 165. Plasmid preS1–48-GFP and preS1-21-GFP express the N-terminal 48 aa (MGQNLSTSNPLGFFPDHQLDPAFRANTANPDWDFNPNK DTWPDANKVG) and 21 aa (MGQNLSTSNP LGFFPDHQLD) of preS1 fused with GFP, respectively. Plasmid preS1 d11-15-GFP contains preS1-48 with a deletion of aa 11–15. PT4-mCherry-FasL-preS1-48-blast (PT4-48 for short) and PT4-mCherry-FasL-preS1-21-blast (PT4-21) are plasmids based on the transposon vector PT4/HB (Addgene) (Wang et al., 2017). Sequences of the fusion proteins expressed by the plasmids are shown in Supplementary Table 1.

### Cell culture and transfection

HEK293 and HEK293FT were purchased from ATCC (American Type Culture Collection). HepAD38 was constructed by Seeger Lab (Ladner et al., 1997) and endowed by Professor Hong Tang of Huaxi Hospital, Sichuan University. HepG2-NTCP cells were kindly provided by Prof. Ningshao Xia, Xiamen University. NTCP expression in this cell line is induced in the presence of doxycycline. These cells were maintained in Dulbecco’s modified Eagle medium supplemented with 10% (vol/vol) fetal bovine serum at 37°C under 5% CO_2_. According to the manufacturer’s instructions, cell transfection was performed with Lipo8000 transfection reagent (Beyotime, China).

### Construction of random peptide library

Based on PT4-21, 7 random amino acids (NNK) were added downstream of the preS1-21 sequence. Briefly, a fragment was amplified from PT4-21 with the primers F1-NNK7tcca′21 (5′-TCGTCTCTTCCANNKNNKNNKNNKNNKNNKNNKTCC GGCGCAACAAACTTCTCTCTGCTGAAAC-3′) and R1-preS 21tgga (5′-TCGTCTCTTGGATCCAACTGGTGGTCGGGAA AGAA-3′). This fragment was then self-ligated by the Golden Gate Cloning method (Engler and Marillonnet, 2014). The 10-μl reaction system contains 100 ng of the fragment, 1 μM ATP, 1 μM DTT, 1 μl Tango buffer (Thermo Fisher Scientific, United States), 0.75 μl BsmBIv2 (NEB, United States), and 0.25 μl T7 DNA ligase (NEB, United States). The reaction parameters were as follows: 30 cycles of 42°C for 5 min and 16°C for 5 min, and 60°C for 5 min. Fifteen such reactions were performed, and then the 150 μl ligation product was transformed into DH5α competent cells. The transformed cells were transferred into 100 ml LB medium and vigorously shaken at 37°C for 1 h. Ampicillin was then added to a final concentration of 100 μg/ml. After shaking for 12 h, the plasmid was extracted and sequenced.

### Construction of stable cell lines by lentivirus transduction

For lentivirus preparation, Lenti-NTCP-GFP-2A-Blast and Lenti-NTCP-mCherry-2A-Blast were co-transfected with psPAX2 and Pmd 2.0G into 293FT cells cultured in 10-cm plates with Lipo8000 transfection reagent (Beyotime, China), respectively. The culture media were harvested at 48 and 72 h after transfection and concentrated by PEG-itTM Virus Precipitation Solution (5x) (SBI) before being resuspended with Opti-MEM. HEK293 cells were seeded in a 6-well plate for lentivirus transduction, and 300 μl lentivirus and 2 μl polybrene were added to the medium. Two days later, the cells were subcultured to a 10 cm-diameter dish, and blasticidin (10 μg/ml) was used to select the cells.

### Construction of stable cell lines with the transposon system

Constructs based on the transposon vector PT4 were co-transfected with plasmid SB100X into HEK293 cells with PT4:SB100X = 9:1 using Lipo8000 transfection reagent. The culture medium was refreshed after 12 and 48 h post-transfection. Stable cell lines were obtained after blasticidin (10 μg/ml) selection for 2 weeks.

### Library screening

The NTCP-GFP stable cell line cultured in 10-cm diameter dishes was detached by trypsin digestion and resuspended in 1xPBS after centrifugation. The cell suspension was frozen in liquid nitrogen and thawed in a water bath at 37°C alternately for 4 times to lyse the cells. The mixture was centrifuged at 12,000 rpm for 3 min and the supernatant was discarded to remove the cytoplasmic proteins. The precipitation was resuspended in 1xPBS and mechanically sheared by passing a 1-ml syringe needle 10 times. The turbid suspension containing membrane segments was then used to incubate with PT4-21 + 7 cells suspended in 1xPBS at room temperature for 2 h. The cells were sorted by a FACS Aria II (Becton Dickinson, United States). The excitation wavelength of 488 nm and emission wavelength of 530/30 nm were selected to detect green fluorescence, and the excitation wavelength of 488 nm and emission wavelength of 575/25 nm were selected to detect red fluorescence. A sorting gate was set so that 5% of the red-fluorescent cells with the highest green fluorescence intensity could be selected in each round (Supplementary Figure 1). The selected cells were transferred to a 6-well plate as soon as possible after sorting. When the cells have expanded to a 10 cm-diameter plate, the next round of screening will be performed.

### Deep sequencing

According to the manufacturer’s instructions, genomic DNA was extracted from enriched PT4-21 + 7 cells using the QIAamp DNA Mini Kit (QIAGEN, Germany). Fragments that contain the random sequences were amplified with primers F-21 (5′-TCTGGTGGTGGAGGCTCTGG-3′) and R-21 (5′-GTTGCTCTTTC AATGAGGGT-3′) from the genomic DNA samples and were subjected to deep sequencing (Sangon Biotech, China).

### Western blot

The protein supernatant was extracted, denatured by boiling in water for 10 min, and cooled on ice for use. After electrophoresis, the gel was transferred to PVDF membranes and blocked with 5% skimmed milk powder at room temperature for 1 h. The membranes were incubated with either the GFP or actin antibody (Beyotime, China) at 4°C overnight. Bound antibody was revealed by IRDye conjugated secondary antibodies (Licor Biosciences, United States) and visualized using an Odyssey CLx system (Licor Biosciences, United States).

### Confocal microscopy

Cells grown on coverslips were fixed with 4% paraformaldehyde for 1 h at 4°C. Plasmids expressing peptides-GFP were transfected into HEK293 cells. The proteins were obtained by repeating freeze-and-thaw of the transfected cell. These proteins were incubated with NTCP-Cherry cells at 37°C for 1 h, respectively. Next, the cells were treated with 40, 6-diamidino-2-phenylindole (DAPI) for nuclear counterstaining, and the images were captured by using a confocal laser scanning microscope (LEICA DMi8, Germany).

### Hepatitis B virus infection of HepG2-Na^+^-taurocholate cotransporting polypeptide cells

The culture media of HepAD38 cells were collected, and HBV was precipitated by 4% PEG8000 and then resolved in Opti-MEM. The viral titer was determined by measuring HBV DNA with qPCR assays. HepG2-NTCP cells were seeded in collagen-treated 12-well plates at a density of 30% of the bottom area. The next day, HepG2-NTCP cells were inoculated with HBV (1,000 genome equivalents per cell) in 4% PEG-8000 and 2.5% DMSO for 18 h. After inoculation, the cells were rinsed with PBS and replenished with DMEM complete medium containing 2.5% DMSO and 2 μg/ml doxycycline. The culture medium was changed every 2 days, and the secreted HBeAg, HBsAg, and intracellular HBV RNA were detected 5 days post-infection.

### Enzyme-linked immunosorbent assay

According to the manufacturer’s instructions, the secreted HBsAg and HBeAg in the culture medium were detected by HBsAg and HBeAg ELISA kits (Kehua, China).

### Ribonucleic acid extraction, RT-qPCR, and northern blot

Five days post-infection, total intracellular ribonucleic acid (RNA) was extracted from HepG2-NTCP cells using Trizol reagent (TIANGEN, China). RNA samples were quantified by a NanoDrop 2000 spectrophotometer (Thermo Fisher Scientific, United States). For reverse transcription, 1 μg of RNA from each sample was added to the reaction system constructed according to the manufacturer’s instructions (FastKing RT Kit, TIANGEN, China). The cDNA of HBV pgRNA was detected by quantitative PCR with primers F pgRNA (5′-TGTTCAAGCCTCCAAGCTGT-3′) and R pgRNA (5′-GACCTGCCTCGTCGTCTAAC-3′). For northern blotting, the RNA samples were resolved using 1.2% gels containing 2% formaldehyde, and then they were transferred onto nylon membranes (Roche, Germany). The HBV RNAs on the membranes were detected using a DIG northern starter kit (Roche, Germany), following the manufacturer’s protocol.

### Data analysis

All FACS data are analyzed with FlowJo (Becton Dickinson, United States). Dose-effect curve plotting and IC50 calculation were conducted with GraphPad Prism 9 (GraphPad, United States). Statistics were performed with the non-parametric Mann–Whitney *U*-test. A value of *P* < 0.05 was considered significant (*p < 0.05, ^**^p < 0.01). All statistical analyses were performed by using GraphPad Prism 9. Results are expressed as the average of three independent experiments. Data are shown as mean value ± standard error.

## Results

### The strategy of the surface display technology

**FIGURE 1:**
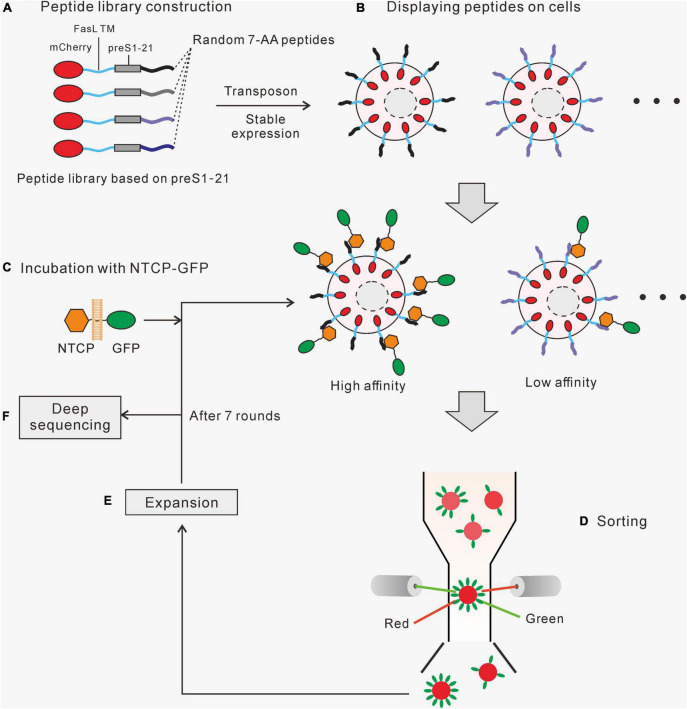
The strategy of the cell surface display system. **(A)** A randomized peptide library is constructed based on preS1 aa2-21. The fusion protein contains a mCherry, a FasL transmembrane domain, a preS1 aa2-21, and a 7-aa randomized peptide. **(B)** A cell library displaying the fusion proteins was created using the Sleeping Beauty transposon system. **(C)** The cell library was incubated with cell membrane segments carrying NTCP-GFP, and those cells expressing high-affinity peptides would bind more NTCP-GFP. **(D)** Red fluorescent (mCherry) cells with relatively higher green fluorescence (GFP) were sorted by flow cytometry. **(E)** The selected cells were amplified before moving on to the next round of the screening process. **(F)** Genomic DNA was extracted from the final enriched cells. Fragments containing target sequences were amplified by PCR and sequenced to identify the enriched peptides.

First, fusion proteins containing mCherry and various peptides (Figure 1A) were stably expressed and displayed on the surface of HEK293 cells using transposon transfection (Figure 1B). Second, the peptide-displaying HEK293 cells were incubated with cell membrane fragments carrying NTCP-GFP, prepared from a cell line stably expressing NTCP-GFP (Figure 1C). Third, cells displaying peptides with a higher affinity for NTCP would bind more NTCP-GFP, which would be selected for further cultivation using flow cytometry (Figures 1D,E). The expanded cells were incubated with NTCP-GFP-expressing membrane fragments for the second round of screening and enrichment. After several rounds of sorting and enrichment, the genomic DNA of the cells was extracted, and the fragments coding different peptides were amplified by PCR for second-generation sequencing to identify their sequences (Figure 1F). Those sequences with the highest abundance and enrichment were investigated further.

### Detection of the interaction between two proteins by surface display

A key strategy is to separate the cells displaying peptides with a high affinity for a target by flow cytometry. To verify the feasibility of this method, we first used the FKBP12-FRB interacting system (Inobe and Nukina, 2016). FKBP12 was fused with mCherry and the FasL transmembrane domain and was displayed on the surface of HEK293 cells. GFP-FRB expressed from HEK293 cells was released by repeated freeze-and-thaw treatment. After that, the GFP-FRB suspension was incubated with mCherry-FasL-FKBP12 cells in the presence or absence of rapamycin. The red-fluorescent cell population drifted up to the area of high green fluorescence in the presence of rapamycin (Supplementary Figure 2), indicating that GFP-FRB binds to mCherry-FasL-FKBP12 cells.

**FIGURE 2:**
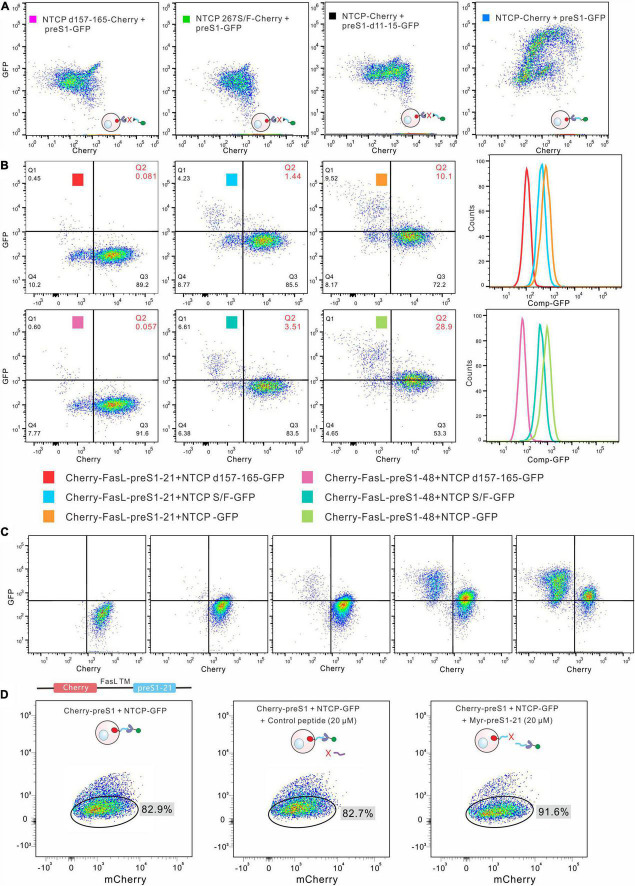
Detection of the interaction between preS1 and NTCP by cell-surface display. **(A)** Detection of preS1-NTCP interaction by the cell-surface display. Wild type NTCP-mCherry was displayed on the cells, and preS1-GFP was used as the bait protein. The interaction was abolished in both the preS1 mutants and the NTCP mutants. **(B)** The preS1 peptides were displayed on the cell surface, and NTCP-GFP on the membrane was used as the bait protein. The mutated NTCP (NTCP d157-165 or 267S/F) lost its affinity for the preS1 peptide, leading to no or low green fluorescence on the Cherry-FasL-preS1-48 cells. The affinity of preS1-48 for NTCP is higher than that of preS1-21, resulting in more green fluorescence on the Cherry-FasL-preS1. **(C)**. Increasing the amount of NTCP-GFP (from left to right) led to increased green fluorescence detected on the Cherry-preS1-21 cells. **(D)**. Competitive assays. Myr-preS1-21s or control peptide was used to compete with preS1-21 on the cell surface.

We next tested whether this method can detect preS1-NTCP interaction. NTCP-mCherry was displayed on the membrane of HEK293 cells, different from mCherry distributed throughout the whole cells (Supplementary Figure 3). PreS1-GFP expressed from HEK293 cells was released by freeze-and-thaw treatment and then incubated with the NTCP-mCherry cells. The FACS results demonstrated that preS1-GFP bound to NTCP-mCherry cells (Figure 2B). In contrast, those cells harboring mutated NTCP (267S/F and d157-165), which have been reported to be deficient in preS1 interaction (Yan et al., 2012; Yang et al., 2016; Uchida et al., 2021), did not show green fluorescence (Figure 2A). Similarly, incubation of NTCP-mCherry cells with a mutated preS1-GFP (preS1-d11-15) resulted in no green fluorescence on the cells (Figure 2A). We also evaluated how well this method works when preS1 peptides (mCherry-FasL-preS1-21 or -48) are presented as a bait protein on the cell surface and NTCP (NTCP-GFP). In this case, NTCP-GFP was expressed in HEK293 cells. Western blotting showed that NTCP-GFP can be detected both on the cellular membrane and in the cytoplasm (Supplementary Figure 4). The membrane segments were used to incubate with mCherry-FasL-preS1 cells. As shown in Figure 2B, flow cytometry can identify the interaction between wild-type NTCP-GFP and mCherry-FasL-preS1. When NTCP is mutated (d157-165), the binding between the two molecules disappears. Moreover, wild-type NTCP-GFP bound more mCherry-FasL-preS1-48 than mCherry-FasL-preS1-21, in line with the fact that preS1-48 has a higher affinity to NTCP than preS1-21.

We tested the method further by gradually increasing the amounts of NTCP-GFP (membrane segments) in the systems. As shown in Figure 2C, more green fluorescence was detected on the mCherry-FasL-preS1-21 cells as NTCP-GFP increased. A synthesized Myr-preS1-21 peptide was used to compete for the binding between mCherry-FasL-preS1-21 cells and NTCP-GFP. As shown in Figure 2D, Myr-preS1-21 increased the proportion of cells that did not bind to NTCP-GFP, while the control peptide did not affect the interaction. Together, the data suggest that FACS can be used to monitor the binding of a bait protein to the proteins on the surface of HEK293 cells.

### Construction and screening of the random peptide library

**FIGURE 3:**
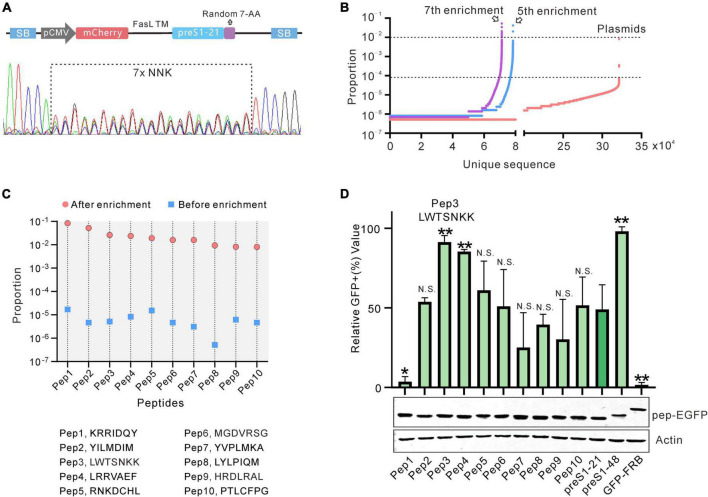
Construction and screening of the random peptide library. **(A)** Sanger sequencing results of the library (PT4-21 + 7). **(B)** Deep sequencing results of the initial or enriched sequence. The number of unique sequences (*x*-axis) and the proportion of each sequence (*y*-axis) are shown. **(C)** The 10 sequences with the highest enrichment fold and proportion after 7 rounds of screening. **(D)** Evaluation of the 10 sequences selected by flow cytometry. The peptides fused with GFP were expressed by HEK293 cells and captured by the cells displaying NTCP-mCherry. Data are shown as means ± SD. Statistical significance was determined using the non-parametric Mann–Whitney *U*-test (*p < 0.05, **p < 0.01).

Seven randomized amino acids were introduced downstream of the preS1-21 peptide (Figure 3A). The detailed process of constructing the library is described in section “Materials and Methods.” Sequencing of the pooled plasmids showed overlapping peaks at the randomized sites (Figure 3A). Deep sequencing detected over 300,000 unique sequences in the pool (Figure 3B). The pooled plasmids (containing transposon sequences) were co-transfected into HEK293 cells with a plasmid expressing the Sleeping Beauty transposase (SB100X). After antibiotic selection, the cells that survived were pooled and subjected to 7 rounds of screening and enrichment. After 5 and 7 rounds of enrichment, the genomic DNA from the cells was extracted, and the fragments containing the randomized sequences were amplified by PCR. Deep sequencing revealed that the diversities of the 2 fragments (the 5th round and 7th round) decreased to less than 80,000, and the unique sequences of the 7th round are even less than that of the 5th round. Compared with the initial library, the number of sequences with a proportion over 10^–4^ significantly increased after the 5th and 7th screening. These results indicated that the sequences were successfully enriched after several rounds of screening.

We chose sequences from the 7th round of enrichment that met the following two criteria for further testing: (1) the proportion of the sequences ranking in the top 10; (2) the enrichment fold of the sequence (the proportion of a sequence in the enrichment library/the proportion of a sequence in the initial Library) ranking in the top 10 as compared with the initial library. Based on these criteria, 10 candidate sequences were selected, named pep1–10. The highest proportion of these sequences (pep1) is close to 10% and the enrichment fold of all the sequences is more than 1,000 times (Figure 3C). These 10 sequences were then evaluated further. We used GFP-fused peptides as bait proteins to coincubate with cells displaying NTCP-mCherry on its surface to discriminate the screening arrangement. This arrangement successfully distinguished GFP-FRB (negative control), preS1-21-GFP, and preS1–48-GFP (Supplementary Figure 5). We used these 10 sequences to construct preS1-21-pep-GFP plasmids. These plasmids were transfected into HEK293 cells, respectively, and the expressed proteins were collected from the supernatant of cell lysis following repeated freeze–thaw treatment. Western blot results showed that the expression level of the fusion proteins (preS1-21-pep-GFP) in each sample was similar (Figure 3D). These proteins were incubated with a stable cell line expressing NTCP-mCherry established with lentivirus transduction and then analyzed by flow cytometry. Compared to the original preS1-21, the affinity of sequence preS1-21-pep3 (preS1-21-LWTSNKK) for NTCP-mCherry cells improved the most, approaching preS1-48 (Figure 3D). Therefore, we selected preS1-21-pep3 for further study.

### Effects of combining pep3 with different genotypes of preS1-21

**FIGURE 4:**
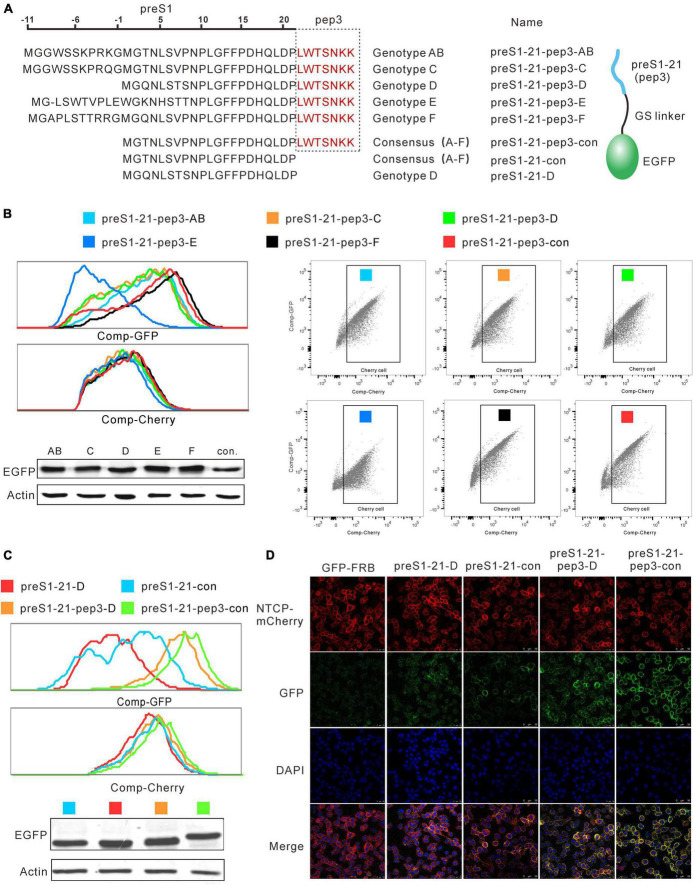
Effects of combining pep3 with different genotypes of preS1-21. **(A)** Plasmids expressing pep3-GFP fused with preS1-21 from genotype A to F and the consensus sequence of A to F were constructed for the evaluation. **(B)** Evaluation of the binding capability for NTCP of pep3 combined with preS1-21 from different genotypes. preS1-21-pep3-F and preS1-21-pep3-con showed the highest binding capability whereas preS1-21-pep3-E the lowest. Western blot was used to check the expression levels of different proteins. **(C)** Evaluation of preS1-21-pep3-con’s ability to bind NTCP. PreS1-21-pep3-con showed a higher binding capacity than preS1-21-D, and preS1-21-pep3-con demonstrated a higher binding capacity than preS1-21-pep3-D. Notably, compared to preS1-21-D and preS1-21-con, both preS1-21-pep3-D and preS1-21-pep3-con showed a much higher binding capability. **(D)** Evaluation of the binding ability by confocal microscopy. preS1-21-pep3-con and preS1-21-pep3-D displayed an apparently higher binding to the cell membrane (green fluorescence) than preS1-21-con and preS1-21-D, while the negative control (GFP-FRB) had only scatter background.

Pep3 was selected based on genotype D preS1-21. We asked whether the preS1-21 sequences from other genotypes would affect the peptides’ affinity for NTCP. To this end, plasmids were constructed to express pep3-GFP fused with preS1-21 from genotype A to F and the consensus sequence of A to F, respectively (Figure 4A). These plasmids were named preS1-21-pep3-AB (genotype A and B have the same sequence), preS1-21-pep3-C, preS1-21-pep3-D, preS1-21-pep3-E, preS1-21-pep3-F, and preS1-21-pep3-con (Figure 4A). The plasmids were transfected into HEK293 cells, and the expressed proteins were collected from the supernatant of the cell lysates following repeated freeze–thaw treatment. The expression levels of these proteins were similar according to the western blot results (Figure 4B). These proteins were used to incubate, respectively, with the stable cell line expressing NTCP-mCherry, and then the results were evaluated using flow cytometry. As shown in Figure 4B, preS1-21-pep3-F and preS1-21-pep3-con displayed the highest binding, whereas preS1-21-pep3-E the lowest. We chose preS1-21-pep3-con for the following experiments due to its smaller size compared with preS121-pep3- F.

We further evaluated preS1-21-pep3-con’s ability to bind NTCP in comparison to a number of different peptides. Cell lysates of preS1-21-pep3-con, preS1-21-pep3-D, preS1-21-con, preS1-21-D were incubated with NTCP-mCherry cells, and the green fluorescence intensity on the cells was used to reflect the binding capacity. As shown in Figure 4C, preS1-21-con showed a higher binding capacity than preS1-21-D and preS1-21-pep3-con demonstrated a higher binding capacity than preS1-21-pep3-D, in line with the results above that the A–F consensus sequence is superior to genotype D in NTCP binding. Notably, compared to preS1-21-D and preS1-21-con, both preS1-21-pep3-D and preS1-21-pep3-con showed a much higher binding capability. These findings were further confirmed by observing the binding with confocal microscopy. As seen in Figure 4D, with comparable NTCP-Cherry expression levels on the cell surface (red fluorescence), preS1-21-pep3-con and preS1-21-pep3-D displayed an apparently higher binding to the cell membrane (green fluorescence) than preS1-21-con and preS1-21-D, while the negative control (GFP-FRB) had only scatter background. These results indicated that pep3 improves the NTCP-binding capability of preS1-21, and that pep3 combined with the consensus preS1-21 is the most effective combination.

### Evaluation of preS1-21-pep3 on HepG2-Na^+^-taurocholate cotransporting polypeptide cell model

**FIGURE 5:**
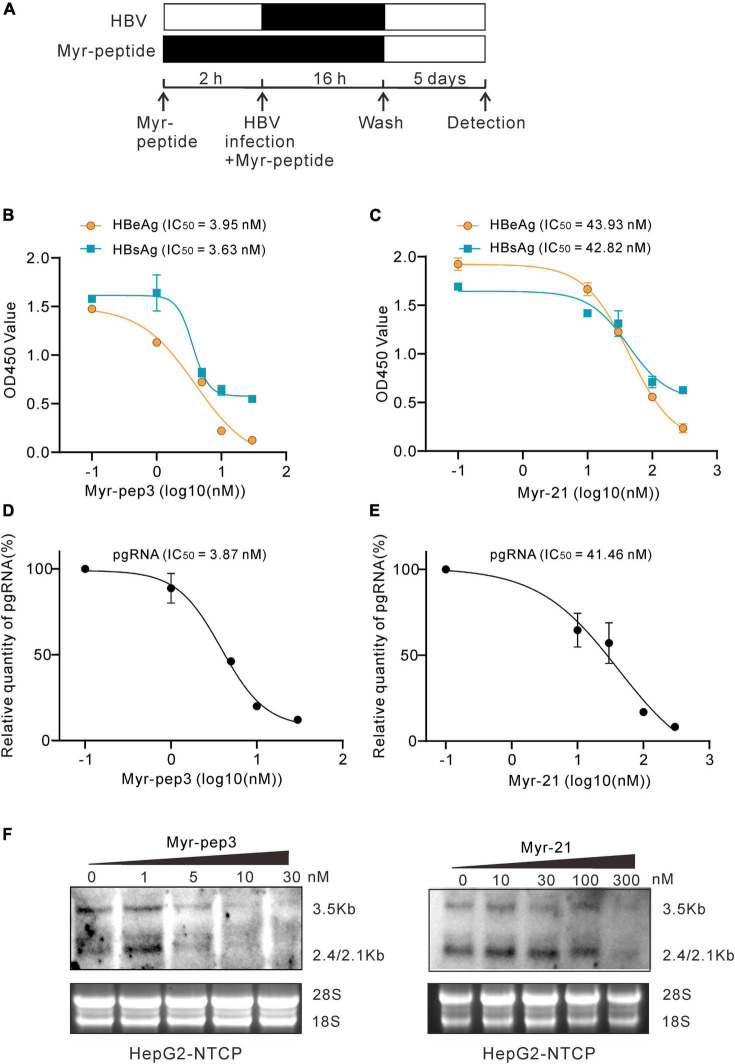
Evaluation of preS1-21-pep3 on HepG2-NTCP cell model. **(A)** Procedure for the testing of the Myr-peptides (Myr-pep3 or Myr-21) on HepG2-NTCP cell model. HepG2-NTCP cells were pretreated with the Myr-peptides for 2 h and then inoculated with HBV for 16 h. The final concentrations were 0, 1, 5, 10, and 30 nM for Myr-pep3, and 0, 10, 30, 100, and 300 nM for Myr-21. After washing out the free HBV and the Myr-peptides, the cells were cultured with the medium in the absence of Myr-peptide for additional 5 days. **(B,C)** Secreted HBeAg and HBsAg in the medium were detected by ELISA, and the IC50s were calculated from the dose-effect curves. **(D,E)** RT-QPCR assay for intracellular HBV pgRNA. Intracellular HBV RNA samples were extracted from the cells and HBV pgRNA was detected by QPCR after reverse transcription. **(F)** Intracellular HBV RNA assays by northern blot. A part of the intracellular HBV RNA samples from **(D,E)** were detected by the northern blot.

The N-Myristoylated peptide preS1-21-pep3-con (Myr-pep3 for short) and the control polypeptide preS1-21-con (Myr-21 for short) were synthesized. The peptides of different concentrations were used to pretreat HepG2-NTCP cells for 2 h before and during the inoculation of HBV. On day 5 after infection, secreted HBeAg and HBsAg were detected by enzyme-linked immunosorbent assay (ELISA), and intracellular HBV RNA and pgRNA were assayed by northern blotting and qPCR (Figure 5A). According to the effect-dose curves, Myr-pep3 demonstrated IC50s of 3.95 nM (HBeAg), 3.63 nM (HBsAg), and 3.87 nM (pgRNA), approximately 10 times more potent than Myr-21 (Figures 5B–E). The northern blotting also showed similar results (Figure 5F).

## Discussion

This study developed a cell display system to screen and optimize NTCP-binding peptides. Compared with the regularly used screening system, this technology has the following advantages. First, by using human cells to display peptides, modifications such as N-myristic acylation and N-glycosylation modification could be easily accomplished. Peptides expressed by bacteriophage, bacteria, or yeast may be modified differently than those expressed by human cells, affecting the binding of peptides to target proteins. Second, the entire receptor protein used as a bait molecule remains on the membrane, preserving its original structure and function, particularly useful for a receptor-like NTCP that lacks a separate extracellular domain. Although *in vitro* protein expression techniques can be used to express NTCP (Fogeron et al., 2017), the proteins produced are not membrane-bound. This must result in a structure that differs from the natural state. Third, the peptides and the bait protein were fused with fluorescent proteins, allowing for convenient and cost-effective labeling for screening. Fourth, the peptides or bait protein can be displayed on the cell surface, allowing cross-validation after screening. The peptides must be displayed on the cell surface to couple the phenotype (binding) with the genotype (sequence) for screening. The bait protein (receptor) can be displayed on the cell surface for validation after enrichment, mimicking the natural condition during therapy. This cross-validation would help to reduce false positivity. Indeed, in our cross-validation, 3 of the 10 peptides were less effective than the original one, demonstrating the procedure’s necessity.

Among the 10 peptides selected after 7 rounds of screening, the one with the highest enrichment degree was inferior to the original peptide in cross-validation, implying that the preliminary screening has false positivity. Several factors could cause false positivity. First, some cells may carry multiple peptide sequences due to multiple sequence integration by the transposon. In this case, a peptide with a low affinity for NTCP can be enriched alongside a peptide with a high affinity for it on the same cells. Second, the membrane fragments obtained by repeated freeze-and-thaw contain other membrane proteins and the target proteins. Consequently, peptides that can bind to other membrane proteins may be enriched. Another disadvantage of the system is that the effective sequences in the initial library are limited. Although more than 300,000 unique sequences were identified in the library, the sequence variety is still much lower than the theoretical value. This problem might be addressed by improving cloning efficiency. In addition, it should be noted that 3% of “NNK” encode a stop codon. The relevant sequences would stop expressing and displaying a result.

Seven rounds of screening with the system led to the identification of the peptide pep3. This peptide was 3,700-fold enriched and represented approximately 2.6% of the total sequences after enrichment. Combining pep3 with a consensus preS1-21 or one of the 5 HBV genotypes revealed that genotype F and the consensus preS1-21 provide the best performance (Figure 4B). The consensus sequence is 11 amino acids shorter than its genotype F equivalent (Figure 4A). Further testing with confocal microscopy confirmed that preS1-21 of genotype D and the consensus both had improved NTCP-binding attributable to pep3. In entry-inhibiting assays, pep3 greatly increased the activity of Myr-21, as evidenced by the fact that it has a 10-fold lower IC50 (3.95 nM, HBeAg) than Myr-21. Given that Myr-pep3 is much smaller than Hepludex (27 aa vs. 47 aa), it makes sense that it must be less immunogenic. Although its IC50 is still greater than Hepludex (IC50 < 1 nm) (Petersen et al., 2008), we believe that additional screening with a 7-random-aa extension could provide novel peptides with equal efficacy to Hepludex and lower molecular weight (34 aa).

In conclusion, a new system for screening NTCP-binding peptides was developed in this study, and a 27-aa peptide with a strong NTCP-binding capability was identified. This research could guide the development of pharmaceutical peptides targeting other viral receptors.

## Data availability statement

The deep sequencing data presented in this study are deposited in NCBI, accession number PRJNA863717.

## Author contributions

P-yW, XY, LG, Y-wW, W-lZ, Y-xS, JL, C-yG, S-yF, J-jL, and S-yL contributed to the implementation of the research. J-LH, P-yW, and A-lH contributed to the design of the research, the analysis of the results, and the writing of the manuscript. All authors contributed to the article and approved the submitted version.
