# Supplementary material for: Establishment of a human cell line with a surface display system for screening and optimizing Na+-taurocholate cotransporting polypeptide-binding peptides
Source: Front Microbiol. 2022 Aug 17;13:920280. doi: 10.3389/fmicb.2022.920280 (PMC9428559; doi:10.3389/fmicb.2022.920280)
Supplement: Supplementary file 1 [file Table_1.DOCX]

**Supplementary Data**

Supplementary Table

| **Plasmid** | **Sequence** | **Color codes** |
| --- | --- | --- |
| NTCP-Cherry | ATGGAGGCCCACAACGCGTCTGCCCCATTCAACTTCACCCTGCCACCCAACTTTGGCAAG  CGCCCCACAGACCTGGCACTGAGCGTCATCCTGGTGTTCATGTTGTTCTTCATCATGCTC  TCGCTGGGCTGCACCATGGAGTTCAGCAAGATCAAGGCTCACTTATGGAAGCCTAAAGGG  CTGGCCATCGCCCTGGTGGCACAGTATGGCATCATGCCCCTCACGGCCTTTGTGCTGGGC  AAGGTCTTCCGGCTGAAGAACATTGAGGCACTGGCCATCTTGGTCTGTGGCTGCTCACCT  GGAGGGAACCTGTCCAATGTCTTCAGTCTGGCCATGAAGGGGGACATGAACCTCAGCATC  GTGATGACCACCTGCTCCACCTTCTGTGCCCTTGGCATGATGCCTCTCCTCCTGTACATC  TACTCCAGGGGGATCTATGATGGGGACCTGAAGGACAAGGTGCCCTATAAAGGCATCGTG  ATATCACTGGTCCTGGTTCTCATTCCTTGCACCATAGGGATCGTCCTCAAATCCAAACGG  CCACAATACATGCGCTATGTCATCAAGGGAGGGATGATCATCATTCTCTTGTGCAGTGTG  GCCGTCACAGTTCTCTCTGCCATCAATGTGGGGAAGAGCATCATGTTTGCCATGACACCA  CTCTTGATTGCCACCTCCTCCCTGATGCCTTTTATTGGCTTTCTGCTGGGTTATGTTCTC  TCTGCTCTCTTCTGCCTCAATGGACGGTGCAGACGCACTGTCAGCATGGAGACTGGATGC  CAAAATGTCCAACTCTGTTCCACCATCCTCAATGTGGCCTTTCCACCTGAAGTCATTGGA  CCACTTTTCTTCTTTCCCCTCCTCTACATGATTTTCCAGCTTGGAGAAGGGCTTCTCCTC  ATTGCCATATTTTGGTGCTATGAGAAATTCAAGACTCCCAAGGATAAAACAAAAATGATC  TACACAGCTGCCACAACTGAAGAAACAATTCCAGGAGCTCTGGGAAATGGCACCTACAAA  GGGGAGGACTGCTCCCCTTGCACAGCCTGGTCTGGATCAGGCGGTGGCGGTTCAGGAGGG  GTGGCTCAGGCGGAGGAGGTTCCGGTGGCGGCGGCAGTGGTGGTGGAGGCTCTGGTGGTGG AGGCTCTGGAGGCGGAGGTTCAGGAGGTGGTGGATCTGGAGGAGGTGGATCTGTGAGC  AAGGGCGAGGAGGATAACATGGCCATCATCAAGGAGTTCATGCGCTTCAAGGTGCACATG  GAGGGCTCCGTGAACGGCCACGAGTTCGAGATCGAGGGCGAGGGCGAGGGCCGCCCCTAC  GAGGGCACCCAGACCGCCAAGCTGAAGGTGACCAAGGGTGGCCCCCTGCCCTTCGCCTGG  GACATCCTGTCCCCTCAGTTCATGTACGGCTCCAAGGCCTACGTGAAGCACCCCGCCGAC  ATCCCCGACTACTTGAAGCTGTCCTTCCCCGAGGGCTTCAAGTGGGAGCGCGTGATGAAC  TTCGAGGACGGCGGCGTGGTGACCGTGACCCAGGACTCCTCCCTGCAGGACGGCGAGTTC  ATCTACAAGGTGAAGCTGCGCGGCACCAACTTCCCCTCCGACGGCCCCGTAATGCAGAAG  AAGACCATGGGCTGGGAGGCCTCCTCCGAGCGGATGTACCCCGAGGACGGCGCCCTGAAG  GGCGAGATCAAGCAGAGGCTGAAGCTGAAGGACGGCGGCCACTACGACGCTGAGGTCAAG  ACCACCTACAAGGCCAAGAAGCCCGTGCAGCTGCCCGGCGCCTACAACGTCAACATCAAG  TTGGACATCACCTCCCACAACGAGGACTACACCATCGTGGAACAGTACGAACGCGCCGAG  GGCCGCCACTCCACCGGCGGCATGGACGAGCTGTACAAG | Green:NTCP  Blue:5G4S  Red:Cherry |
| NTCP-GFP-2A-Blast | ATGGAGGCCCACAACGCGTCTGCCCCATTCAACTTCACCCTGCCACCCAACTTTGGCAAG  CGCCCCACAGACCTGGCACTGAGCGTCATCCTGGTGTTCATGTTGTTCTTCATCATGCTC  TCGCTGGGCTGCACCATGGAGTTCAGCAAGATCAAGGCTCACTTATGGAAGCCTAAAGGG  CTGGCCATCGCCCTGGTGGCACAGTATGGCATCATGCCCCTCACGGCCTTTGTGCTGGGC  AAGGTCTTCCGGCTGAAGAACATTGAGGCACTGGCCATCTTGGTCTGTGGCTGCTCACCT  GGAGGGAACCTGTCCAATGTCTTCAGTCTGGCCATGAAGGGGGACATGAACCTCAGCATC  GTGATGACCACCTGCTCCACCTTCTGTGCCCTTGGCATGATGCCTCTCCTCCTGTACATC  TACTCCAGGGGGATCTATGATGGGGACCTGAAGGACAAGGTGCCCTATAAAGGCATCGTG  ATATCACTGGTCCTGGTTCTCATTCCTTGCACCATAGGGATCGTCCTCAAATCCAAACGG  CCACAATACATGCGCTATGTCATCAAGGGAGGGATGATCATCATTCTCTTGTGCAGTGTG  GCCGTCACAGTTCTCTCTGCCATCAATGTGGGGAAGAGCATCATGTTTGCCATGACACCA  CTCTTGATTGCCACCTCCTCCCTGATGCCTTTTATTGGCTTTCTGCTGGGTTATGTTCTC  TCTGCTCTCTTCTGCCTCAATGGACGGTGCAGACGCACTGTCAGCATGGAGACTGGATGC  CAAAATGTCCAACTCTGTTCCACCATCCTCAATGTGGCCTTTCCACCTGAAGTCATTGGA  CCACTTTTCTTCTTTCCCCTCCTCTACATGATTTTCCAGCTTGGAGAAGGGCTTCTCCTC  ATTGCCATATTTTGGTGCTATGAGAAATTCAAGACTCCCAAGGATAAAACAAAAATGATC  TACACAGCTGCCACAACTGAAGAAACAATTCCAGGAGCTCTGGGAAATGGCACCTACAAA  GGGGAGGACTGCTCCCCTTGCACAGCCTGGTCTGGATCAGGCGGTGGCGGTTCAGGAGGT  GGTGGCTCAGGCGGAGGAGGTTCCGGTGGCGGCGGCAGTGGTGGTGGAGGCTCTGGTGGT  GGAGGCTCTGGAGGCGGAGGTTCAGGAGGTGGTGGATCTGGAGGAGGTGGATCTGTGGTG  AGCAAGGGCGAGGAGCTGTTCACCGGGGTGGTGCCCATCCTGGTCGAGCTGGACGGCGAC  GTAAACGGCCACAAGTTCAGCGTGTCCGGCGAGGGCGAGGGCGATGCCACCTACGGCAAG  CTGACCCTGAAGTTCATCTGCACCACCGGCAAGCTGCCCGTGCCCTGGCCCACCCTCGTG  ACCACCCTGACCTACGGCGTGCAGTGCTTCAGCCGCTACCCCGACCACATGAAGCAGCAC  GACTTCTTCAAGTCCGCCATGCCCGAAGGCTACGTCCAGGAGCGCACCATCTTCTTCAAG  GACGACGGCAACTACAAGACCCGCGCCGAGGTGAAGTTCGAGGGCGACACCCTGGTGAAC  CGCATCGAGCTGAAGGGCATCGACTTCAAGGAGGACGGCAACATCCTGGGGCACAAGCTG  GAGTACAACTACAACAGCCACAACGTCTATATCATGGCCGACAAGCAGAAGAACGGCATC  AAGGTGAACTTCAAGATCCGCCACAACATCGAGGACGGCAGCGTGCAGCTCGCCGACCAC  TACCAGCAGAACACCCCCATCGGCGACGGCCCCGTGCTGCTGCCCGACAACCACTACCTG  AGCACCCAGTCCGCCCTGAGCAAAGACCCCAACGAGAAGCGCGATCACATGGTCCTGCTG  GAGTTCGTGACCGCCGCCGGGATCACTCTCGGCATGGACGAGCTGTACAAGGGATCCGGC  GCAACAAACTTCTCTCTGCTGAAACAAGCCGGAGATGTCGAAGAGAATCCTGGACCGATG  GCCAAGCCTTTGTCTCAAGAAGAATCCACCCTCATTGAAAGAGCAACGGCTACAATCAAC  AGCATCCCCATCTCTGAAGACTACAGCGTCGCCAGCGCAGCTCTCTCTAGCGACGGCCGC  ATCTTCACTGGTGTCAATGTATATCATTTTACTGGGGGACCTTGTGCAGAACTCGTGGTG  CTGGGCACTGCTGCTGCTGCGGCAGCTGGCAACCTGACTTGTATCGTCGCGATCGGAAAT  GAGAACAGGGGCATCTTGAGCCCCTGCGGACGGTGCCGACAGGTGCTTCTCGATCTGCAT  CCTGGGATCAAAGCCATAGTGAAGGACAGTGATGGACAGCCGACGGCAGTTGGGATTCGT  GAATTGCTGCCCTCTGGTTATGTGTGGGAGGGCTAA | Green :NTCP  Blue:GS Linker  Black:GFP  Purple:2A  Black:Blast |
| Cherry-fasL-PreS1 48-2A-Blast | ATGGTGAGCAAGGGCGAGGAGGATAACATGGCCATCATCAAGGAGTTCATGCGCTTCAAG  GTGCACATGGAGGGCTCCGTGAACGGCCACGAGTTCGAGATCGAGGGCGAGGGCGAGGGC  CGCCCCTACGAGGGCACCCAGACCGCCAAGCTGAAGGTGACCAAGGGTGGCCCCCTGCCC  TTCGCCTGGGACATCCTGTCCCCTCAGTTCATGTACGGCTCCAAGGCCTACGTGAAGCAC  CCCGCCGACATCCCCGACTACTTGAAGCTGTCCTTCCCCGAGGGCTTCAAGTGGGAGCGC  GTGATGAACTTCGAGGACGGCGGCGTGGTGACCGTGACCCAGGACTCCTCCCTGCAGGAC  GGCGAGTTCATCTACAAGGTGAAGCTGCGCGGCACCAACTTCCCCTCCGACGGCCCCGTA  ATGCAGAAGAAGACCATGGGCTGGGAGGCCTCCTCCGAGCGGATGTACCCCGAGGACGGC  GCCCTGAAGGGCGAGATCAAGCAGAGGCTGAAGCTGAAGGACGGCGGCCACTACGACGCT  AACATCAAGTTGGACATCACCTCCCACAACGAGGACTACACCATCGTGGAACAGTACGAA  CGCGCCGAGGGCCGCCACTCCACCGGCGGCATGGACGAGCTGTACAAGGGCGGT GGCGG  TTCAA AGAAGAGAGG GAACCACAGCACAGGCCTGTGTCTCCTTGTGATGTTTTTCATG  GTTCTGGTTGCCTTGGTAGGATTGGGCCTGGGGATGTTTCAGCTCTTCCACCTACAGAAG  GAGTCGTCTGGATCAGGCGGTGGCGGTTCAGGAGGTGGTGGCTCAGGCGGAGGAGGTTCC  GGTGGCGGCGGCAGTGGTGGTGGAGGCTCTGGTGGTGGAGGCTCTGGAGGCGGAGGTTCA  GGAGGTGGTGGATCTGGAGGAGGTGGATCTGGGCAGAATCTTTCCACCAGCAATCCTCTG  GGATTCTTTCCCGACCACCAGTTGGATCCAGCCTTCAGAGCAAACACCGCAAATCCAGAT  TGGGACTTCAATCCCAACAAGGACACCTGGCCAGACGCCAACAAGGTAGGAGGATCCGGC  GCAACAAACTTCTCTCTGCTGAAACAAGCCGGAGATGTCGAAGAGAATCCTGGACCGATG  GCCAAGCCTTTGTCTCAAGAAGAATCCACCCTCATTGAAAGAGCAACGGCTACAATCAAC  AGCATCCCCATCTCTGAAGACTACAGCGTCGCCAGCGCAGCTCTCTCTAGCGACGGCCGC  ATCTTCACTGGTGTCAATGTATATCATTTTACTGGGGGACCTTGTGCAGAACTCGTGGTG  CTGGGCACTGCTGCTGCTGCGGCAGCTGGCAACCTGACTTGTATCGTCGCGATCGGAAAT  GAGAACAGGGGCATCTTGAGCCCCTGCGGACGGTGCCGACAGGTGCTTCTCGATCTGCAT  CCTGGGATCAAAGCCATAGTGAAGGACAGTGATGGACAGCCGACGGCAGTTGGGATTCGT  GAATTGCTGCCCTCTGGTTATGTGTGGGAGGGCTAA | Red:Cherry  Yellow：FasL  Blue:GS Linker  Green:PreS1 48  Purple:2A  Black:Blast |
| Cherry-fasL-PreS1 21-2A-Blast | ATGGTGAGCAAGGGCGAGGAGGATAACATGGCCATCATCAAGGAGTTCATGCGCTTCAAG  GTGCACATGGAGGGCTCCGTGAACGGCCACGAGTTCGAGATCGAGGGCGAGGGCGAGGGC  CGCCCCTACGAGGGCACCCAGACCGCCAAGCTGAAGGTGACCAAGGGTGGCCCCCTGCCC  TTCGCCTGGGACATCCTGTCCCCTCAGTTCATGTACGGCTCCAAGGCCTACGTGAAGCAC  CCCGCCGACATCCCCGACTACTTGAAGCTGTCCTTCCCCGAGGGCTTCAAGTGGGAGCGC  GTGATGAACTTCGAGGACGGCGGCGTGGTGACCGTGACCCAGGACTCCTCCCTGCAGGAC  GGCGAGTTCATCTACAAGGTGAAGCTGCGCGGCACCAACTTCCCCTCCGACGGCCCCGTA  ATGCAGAAGAAGACCATGGGCTGGGAGGCCTCCTCCGAGCGGATGTACCCCGAGGACGGC  GCCCTGAAGGGCGAGATCAAGCAGAGGCTGAAGCTGAAGGACGGCGGCCACTACGACGCT  AACATCAAGTTGGACATCACCTCCCACAACGAGGACTACACCATCGTGGAACAGTACGAA  CGCGCCGAGGGCCGCCACTCCACCGGCGGCATGGACGAGCTGTACAAGGGCGGT GGCGG  TTCAA AGAAGAGAGG GAACCACAGCACAGGCCTGTGTCTCCTTGTGATGTTTTTCATG  GTTCTGGTTGCCTTGGTAGGATTGGGCCTGGGGATGTTTCAGCTCTTCCACCTACAGAAG  GAGTCGTCTGGATCAGGCGGTGGCGGTTCAGGAGGTGGTGGCTCAGGCGGAGGAGGTTCC  GGTGGCGGCGGCAGTGGTGGTGGAGGCTCTGGTGGTGGAGGCTCTGGAGGCGGAGGTTCA  GGAGGTGGTGGATCTGGAGGAGGTGGATCTGGGCAGAATCTTTCCACCAGCAATCCTCTG  GGATTCTTTCCCGACCACCAGTTGGATCCAGGATCCGGCGCAACAAACTTCTCTCTGCTG  AAACAAGCCGGAGATGTCGAAGAGAATCCTGGACCGATGGCCAAGCCTTTGTCTCAAGAA  GAATCCACCCTCATTGAAAGAGCAACGGCTACAATCAACAGCATCCCCATCTCTGAAGAC  TACAGCGTCGCCAGCGCAGCTCTCTCTAGCGACGGCCGCATCTTCACTGGTGTCAATGTA  TATCATTTTACTGGGGGACCTTGTGCAGAACTCGTGGTGCTGGGCACTGCTGCTGCTGCG  GCAGCTGGCAACCTGACTTGTATCGTCGCGATCGGAAATGAGAACAGGGGCATCTTGAGC  CCCTGCGGACGGTGCCGACAGGTGCTTCTCGATCTGCATCCTGGGATCAAAGCCATAGTG  AAGGACAGTGATGGACAGCCGACGGCAGTTGGGATTCGTGAATTGCTGCCCTCTGGTTAT  GTGTGGGAGGGCTAA | Red:Cherry  Yellow：FasL  Blue:GS Linker  Green:PreS1 48  Purple:2A  Black:Blast |
| FRB-GFP | ATGGTGATCCTCTGGCATGAGATGTGGCATGAAGGCCTGGAAGAGGCATCTCGTTTGTAC  TTTGGGGAAAGGAACGTGAAAGGCATGTTTGAGGTGCTGGAGCCCTTGCATGCTATGATG  GAACGGGGCCCCCAGACTCTGAAGGAAACATCCTTTAATCAGGCCTATGGTCGAGATTTA  ATGGAGGCCCAAGAGTGGTGCAGGAAGTACATGAAATCAGGGAATGTCAAGGACCTCCTC  CAAGCCTGGGACCTTCTATTATTCAATTGTCGTCTGGATCAGGCGTGGCGGTTCAGGAGG  TGGTGGCTCAGGCGGAGGAGGTTCCGGTGGCGGCGGCAGTGGTGGTGGAGGCTCTGGTGG  TGGAGGCTCTGGAGGCGGAGGTTCAGGAGGTGGTGGATCTGGAGGAGGTGGATCTGTGAG  CAAGGGCGAGGAGCTGTTCACCGGGGTGGTGCCCATCCTGGTCGAGCTGGACGGCGACGT  AAACGGCCACAAGTTCAGCGTGTCCGGCGAGGGCGAGGGCGATGCCACCTACGGCAAGCT  GACCCTGAAGTTCATCTGCACCACCGGCAAGCTGCCCGTGCCCTGGCCCACCCTCGTGAC  CACCCTGACCTACGGCGTGCAGTGCTTCAGCCGCTACCCCGACCACATGAAGCAGCACGA  CTTCTTCAAGTCCGCCATGCCCGAAGGCTACGTCCAGGAGCGCACCATCTTCTTCAAGGA  CGACGGCAACTACAAGACCCGCGCCGAGGTGAAGTTCGAGGGCGACACCCTGGTGAACCG  CATCGAGCTGAAGGGCATCGACTTCAAGGAGGACGGCAACATCCTGGGGCACAAGCTGGA  GTACAACTACAACAGCCACAACGTCTATATCATGGCCGACAAGCAGAAGAACGGCATCAA  GGTGAACTTCAAGATCCGCCACAACATCGAGGACGGCAGCGTGCAGCTCGCCGACCACTA  CCAGCAGAACACCCCCATCGGCGACGGCCCCGTGCTGCTGCCCGACAACCACTACCTGAG  CACCCAGTCCGCCCTGAGCAAAGACCCCAACGAGAAGCGCGATCACATGGTCCTGCTGGA  GTTCGTGACCGCCGCCGGGATCACTCTCGGCATGGACGAGCTGTACAAGTAA | Red:Cherry  Blue:GS Linker  Black:GFP |
| GFP-FRB | GTGAGCAAGGGCGAGGAGCTGTTCACCGGGGTGGTGCCCATCCTGGTCGAGCTGGACGGC  GACGTAAACGGCCACAAGTTCAGCGTGTCCGGCGAGGGCGAGGGCGATGCCACCTACGGC  AAGCTGACCCTGAAGTTCATCTGCACCACCGGCAAGCTGCCCGTGCCCTGGCCCACCCTC  GTGACCACCCTGACCTACGGCGTGCAGTGCTTCAGCCGCTACCCCGACCACATGAAGCAG  CACGACTTCTTCAAGTCCGCCATGCCCGAAGGCTACGTCCAGGAGCGCACCATCTTCTTC  AAGGACGACGGCAACTACAAGACCCGCGCCGAGGTGAAGTTCGAGGGCGACACCCTGGTG  AACCGCATCGAGCTGAAGGGCATCGACTTCAAGGAGGACGGCAACATCCTGGGGCACAAG  CTGGAGTACAACTACAACAGCCACAACGTCTATATCATGGCCGACAAGCAGAAGAACGGC  ATCAAGGTGAACTTCAAGATCCGCCACAACATCGAGGACGGCAGCGTGCAGCTCGCCGAC  CACTACCAGCAGAACACCCCCATCGGCGACGGCCCCGTGCTGCTGCCCGACAACCACTAC  CTGAGCACCCAGTCCGCCCTGAGCAAAGACCCCAACGAGAAGCGCGATCACATGGTCCTG  CTGGAGTTCGTGACCGCCGCCGGGATCACTCTCGGCATGGACGAGCTGTACAAGTCGTCT  GGATCAGGCGTGGCGGTTCAGGAGGTGGTGGCTCAGGCGGAGGAGGTTCCGGTGGCGGCG  GCAGTGGTGGTGGAGGCTCTGGTGGTGGAGGCTCTGGAGGCGGAGGTTCAGGAGGTGGTG  GATCTGGAGGAGGTGGATCTATCCTCTGGCATGAGATGTGGCATGAAGGCCTGG  AAGAGGCATCTCGTTTGTACTTTGGGGAAAGGAACGTGAAAGGCATGTTTGAGGTGCTGG  AGCCCTTGCATGCTATGATGGAACGGGGCCCCCAGACTCTGAAGGAAACATCCTTTAATC  AGGCCTATGGTCGAGATTTAATGGAGGCCCAAGAGTGGTGCAGGAAGTACATGAAATCAG  GGAATGTCAAGGACCTCCTCCAAGCCTGGGACCTTCTATTATTCAATTGTAA | Red: Cherry  Blue: GS Linker  Black: GFP |
| PreS1 48-GFP | ATGGGGCAGAATCTTTCCACCAGCAATCCTCTGGGATTCTTTCCCGACCACCAGTTGGAT  CCAGCCTTCAGAGCAAACACCGCAAATCCAGATTGGGACTTCAATCCCAACAAGGACACC  TGGCCAGACGCCAACAAGGTAGGATCGTCTGGATCAGGCGGTGGCGGTTCAGGAGGTGGT  GGCTCAGGCGGAGGAGGTTCCGGTGGCGGCGGCAGTGGTGGTGGAGGCTCTGGTGGTGGA  GGCTCTGGAGGCGGAGGTTCAGGAGGTGGTGGATCTGGAGGAGGTGGATCTGTGAGCAAG  GGCGAGGAGCTGTTCACCGGGGTGGTGCCCATCCTGGTCGAGCTGGACGGCGACGTAAAC  GGCCACAAGTTCAGCGTGTCCGGCGAGGGCGAGGGCGATGCCACCTACGGCAAGCTGACC  CTGAAGTTCATCTGCACCACCGGCAAGCTGCCCGTGCCCTGGCCCACCCTCGTGACCACC  CTGACCTACGGCGTGCAGTGCTTCAGCCGCTACCCCGACCACATGAAGCAGCACGACTTC  TTCAAGTCCGCCATGCCCGAAGGCTACGTCCAGGAGCGCACCATCTTCTTCAAGGACGAC  GGCAACTACAAGACCCGCGCCGAGGTGAAGTTCGAGGGCGACACCCTGGTGAACCGCATC  GAGCTGAAGGGCATCGACTTCAAGGAGGACGGCAACATCCTGGGGCACAAGCTGGAGTAC  AACTACAACAGCCACAACGTCTATATCATGGCCGACAAGCAGAAGAACGGCATCAAGGTG  AACTTCAAGATCCGCCACAACATCGAGGACGGCAGCGTGCAGCTCGCCGACCACTACCAG  CAGAACACCCCCATCGGCGACGGCCCCGTGCTGCTGCCCGACAACCACTACCTGAGCACC  CAGTCCGCCCTGAGCAAAGACCCCAACGAGAAGCGCGATCACATGGTCCTGCTGGAGTTC  GTGACCGCCGCCGGGATCACTCTCGGCATGGACGAGCTGTACAAGTAA | Red: PreS1 48  Blue: GS Linker  Black: GFP |
| PreS1 21-GFP | ATGGGGCAGAATCTTTCCACCAGCAATCCTCTGGGATTCTTTCCCGACCACCAGTTGGAT  CCATCGTCTGGATCAGGCGGTGGCGGTTCAGGAGGTGGTGGCTCAGGCGGAGGAGGTTCC  GGTGGCGGCGGCAGTGGTGGTGGAGGCTCTGGTGGTGGAGGCTCTGGAGGCGGAGGTTCA  GGAGGTGGTGGATCTGGAGGAGGTGGATCTGTGAGCAAGGGCGAGGAGCTGTTCACCGGG  GTGGTGCCCATCCTGGTCGAGCTGGACGGCGACGTAAACGGCCACAAGTTCAGCGTGTCC  GGCGAGGGCGAGGGCGATGCCACCTACGGCAAGCTGACCCTGAAGTTCATCTGCACCACC  GGCAAGCTGCCCGTGCCCTGGCCCACCCTCGTGACCACCCTGACCTACGGCGTGCAGTGC  TTCAGCCGCTACCCCGACCACATGAAGCAGCACGACTTCTTCAAGTCCGCCATGCCCGAA  GGCTACGTCCAGGAGCGCACCATCTTCTTCAAGGACGACGGCAACTACAAGACCCGCGCC  GAGGTGAAGTTCGAGGGCGACACCCTGGTGAACCGCATCGAGCTGAAGGGCATCGACTTC  AAGGAGGACGGCAACATCCTGGGGCACAAGCTGGAGTACAACTACAACAGCCACAACGTC  TATATCATGGCCGACAAGCAGAAGAACGGCATCAAGGTGAACTTCAAGATCCGCCACAAC  ATCGAGGACGGCAGCGTGCAGCTCGCCGACCACTACCAGCAGAACACCCCCATCGGCGAC  GGCCCCGTGCTGCTGCCCGACAACCACTACCTGAGCACCCAGTCCGCCCTGAGCAAAGAC  CCCAACGAGAAGCGCGATCACATGGTCCTGCTGGAGTTCGTGACCGCCGCCGGGATCACT  CTCGGCATGGACGAGCTGTACAAGTAA | Red: PreS1 21  Blue: GS Linker  Black: GFP |
| PreS1 d11-15-GFP | ATGGGGCAGAATCTTTCCACCAGCAATCCTGACCACCAGTTGGATCCAGCCTTCAGAGCA  AACACCGCAAATCCAGATTGGGACTTCAATCCCAACAAGGACACCTGGCCAGACGCCAAC  AAGGTAGGATCGTCTGGATCAGGCGGTGGCGGTTCAGGAGGTGGTGGCTCAGGCGGAGGA  GGTTCCGGTGGCGGCGGCAGTGGTGGTGGAGGCTCTGGTGGTGGAGGCTCTGGAGGCGGA  GGTTCAGGAGGTGGTGGATCTGGAGGAGGTGGATCTGTGAGCAAGGGCGAGGAGCTGTTC  ACCGGGGTGGTGCCCATCCTGGTCGAGCTGGACGGCGACGTAAACGGCCACAAGTTCAGC  GTGTCCGGCGAGGGCGAGGGCGATGCCACCTACGGCAAGCTGACCCTGAAGTTCATCTGC  ACCACCGGCAAGCTGCCCGTGCCCTGGCCCACCCTCGTGACCACCCTGACCTACGGCGTG  CAGTGCTTCAGCCGCTACCCCGACCACATGAAGCAGCACGACTTCTTCAAGTCCGCCATG  CCCGAAGGCTACGTCCAGGAGCGCACCATCTTCTTCAAGGACGACGGCAACTACAAGACC  CGCGCCGAGGTGAAGTTCGAGGGCGACACCCTGGTGAACCGCATCGAGCTGAAGGGCATC  GACTTCAAGGAGGACGGCAACATCCTGGGGCACAAGCTGGAGTACAACTACAACAGCCAC  AACGTCTATATCATGGCCGACAAGCAGAAGAACGGCATCAAGGTGAACTTCAAGATCCGC  CACAACATCGAGGACGGCAGCGTGCAGCTCGCCGACCACTACCAGCAGAACACCCCCATC  GGCGACGGCCCCGTGCTGCTGCCCGACAACCACTACCTGAGCACCCAGTCCGCCCTGAGC  AAAGACCCCAACGAGAAGCGCGATCACATGGTCCTGCTGGAGTTCGTGACCGCCGCCGGG  ATCACTCTCGGCATGGACGAGCTGTACAAGTAA | Red: PreS1 d11-15  Blue: GS Linker  Black: GFP |
| NTCP 267S/F | ATGGAGGCCCACAACGCGTCTGCCCCATTCAACTTCACCCTGCCACCCAACTTTGGCAAG  CGCCCCACAGACCTGGCACTGAGCGTCATCCTGGTGTTCATGTTGTTCTTCATCATGCTC  TCGCTGGGCTGCACCATGGAGTTCAGCAAGATCAAGGCTCACTTATGGAAGCCTAAAGGG  CTGGCCATCGCCCTGGTGGCACAGTATGGCATCATGCCCCTCACGGCCTTTGTGCTGGGC  AAGGTCTTCCGGCTGAAGAACATTGAGGCACTGGCCATCTTGGTCTGTGGCTGCTCACCT  GGAGGGAACCTGTCCAATGTCTTCAGTCTGGCCATGAAGGGGGACATGAACCTCAGCATC  GTGATGACCACCTGCTCCACCTTCTGTGCCCTTGGCATGATGCCTCTCCTCCTGTACATC  TACTCCAGGGGGATCTATGATGGGGACCTGAAGGACAAGGTGCCCTATAAAGGCATCGTG  ATATCACTGGTCCTGGTTCTCATTCCTTGCACCATAGGGATCGTCCTCAAATCCAAACGG  CCACAATACATGCGCTATGTCATCAAGGGAGGGATGATCATCATTCTCTTGTGCAGTGTG  GCCGTCACAGTTCTCTCTGCCATCAATGTGGGGAAGAGCATCATGTTTGCCATGACACCA  CTCTTGATTGCCACCTCCTCCCTGATGCCTTTTATTGGCTTTCTGCTGGGTTATGTTCTC  TCTGCTCTCTTCTGCCTCAATGGACGGTGCAGACGCACTGTCAGCATGGAGACTGGATGC  CAAAATGTCCAACTCTGTTTCACCATCCTCAATGTGGCCTTTCCACCTGAAGTCATTGGA  CCACTTTTCTTCTTTCCCCTCCTCTACATGATTTTCCAGCTTGGAGAAGGGCTTCTCCTC  ATTGCCATATTTTGGTGCTATGAGAAATTCAAGACTCCCAAGGATAAAACAAAAATGATC  TACACAGCTGCCACAACTGAAGAAACAATTCCAGGAGCTCTGGGAAATGGCACCTACAAA  GGGGAGGACTGCTCCCCTTGCACAGCC | Green: NTCP  (267S/F)  Yellow:  267F |
| NTCP d157-165 | ATGGAGGCCCACAACGCGTCTGCCCCATTCAACTTCACCCTGCCACCCAACTTTGGCAAG  CGCCCCACAGACCTGGCACTGAGCGTCATCCTGGTGTTCATGTTGTTCTTCATCATGCTC  TCGCTGGGCTGCACCATGGAGTTCAGCAAGATCAAGGCTCACTTATGGAAGCCTAAAGGG  CTGGCCATCGCCCTGGTGGCACAGTATGGCATCATGCCCCTCACGGCCTTTGTGCTGGGC  AAGGTCTTCCGGCTGAAGAACATTGAGGCACTGGCCATCTTGGTCTGTGGCTGCTCACCT  GGAGGGAACCTGTCCAATGTCTTCAGTCTGGCCATGAAGGGGGACATGAACCTCAGCATC  GTGATGACCACCTGCTCCACCTTCTGTGCCCTTGGCATGATGCCTCTCCTCCTGTACATC  TACTCCAGGGGGATCTATGATGGGGACCTGAAGGACAAGGTGCCCTATGTTCTCATTCCT  TGCACCATAGGGATCGTCCTCAAATCCAAACGGCCACAATACATGCGCTATGTCATCAAG  GGAGGGATGATCATCATTCTCTTGTGCAGTGTGGCCGTCACAGTTCTCTCTGCCATCAAT  GTGGGGAAGAGCATCATGTTTGCCATGACACCACTCTTGATTGCCACCTCCTCCCTGATG  CCTTTTATTGGCTTTCTGCTGGGTTATGTTCTCCAAAATGTCCAACTCTGTTCCACCATC  CTCAATGTGGCCTTTCCACCTGAAGTCATTGGACCACTTTTCTTCTTTCCCCTCCTCTAC  ATGATTTTCCAGCTTGGAGAAGGGCTTCTCCTCATTGCCATATTTTGGTGCTATGAGAAA  TTCAAGACTCCCAAGGATAAAACAAAAATGATCTACACAGCTGCCACAACTGAAGAAACA  ATTCCAGGAGCTCTGGGAAATGGCACCTACAAAGGGGAGGACTGCTCCCCTTGCACAGCC | Green: NTCP  (d157-165) |
| Cherry-fasL-PreS1 21+7-2A-Blast  （PT4-21+7） | ATGGTGAGCAAGGGCGAGGAGGATAACATGGCCATCATCAAGGAGTTCATGCGCTTCAAG  GTGCACATGGAGGGCTCCGTGAACGGCCACGAGTTCGAGATCGAGGGCGAGGGCGAGGGC  CGCCCCTACGAGGGCACCCAGACCGCCAAGCTGAAGGTGACCAAGGGTGGCCCCCTGCCC  TTCGCCTGGGACATCCTGTCCCCTCAGTTCATGTACGGCTCCAAGGCCTACGTGAAGCAC  CCCGCCGACATCCCCGACTACTTGAAGCTGTCCTTCCCCGAGGGCTTCAAGTGGGAGCGC  GTGATGAACTTCGAGGACGGCGGCGTGGTGACCGTGACCCAGGACTCCTCCCTGCAGGAC  GGCGAGTTCATCTACAAGGTGAAGCTGCGCGGCACCAACTCCCCTCCGACGGCCCCGTA  ATGCAGAAGAAGACCATGGGCTGGGAGGCCTCCTCCGAGCGGATGTACCCCGAGGACGGC  GCCCTGAAGGGCGAGATCAAGCAGAGGCTGAAGCTGAAGGACGGCGGCCACTACGACGCT  GAGGTCAAGACCACCTACAAGGCCAAGAAGCCCGTGCAGCTGCCCGGCGCCTACAACGTC  AACATCAAGTTGGACATCACCTCCCACAACGAGGACTACACCATCGTGGAACAGTACGAA  CGCGCCGAGGGCCGCCACTCCACCGGCGGCATGGACGAGCTGTACAAGGGCGGTGGCGGT  TCAAAGAAGAGAGGGAACCACAGCACAGGCCTGTGTCTCCTTGTGATGTTTTTCATGGTT  CTGGTTGCCTTGGTAGGATTGGGCCTGGGGATGTTTCAGCTCTTCCACCTACAGAAGGAG  TCGTCTGGATCAGGCGGTGGCGGTTCAGGAGGTGGTGGCTCAGGCGGAGGAGGTTCCGGT  GGCGGCGGCAGTGGTGGTGGAGGCTCTGGTGGTGGAGGCTCTGGAGGCGGAGGTTCAGGA  GGTGGTGGATCTGGAGGAGGTGGATCTGGGCAGAATCTTTCCACCAGCAATCCTCTGGGA  TTCTTTCCCGACCACCAGTTGGATCCANNKNNKNNKNNKNNKNNKNNKGGATCCGG  CGCAACAAACTTCTCTCTGCTGAAACAAGCCGGAGATGTCGAAGAGAATCCTGGACCGAT  GGCCAAGCCTTTGTCTCAAGAAGAATCCACCCTCATTGAAAGAGCAACGGCTACAATCAA  CAGCATCCCCATCTCTGAAGACTACAGCGTCGCCAGCGCAGCTCTCTCTAGCGACGGCCG  CATCTTCACTGGTGTCAATGTATATCATTTTACTGGGGGACCTTGTGCAGAACTCGTGGT  GCTGGGCACTGCTGCTGCTGCGGCAGCTGGCAACCTGACTTGTATCGTCGCGATCGGAAA  TGAGAACAGGGGCATCTTGAGCCCCTGCGGACGGTGCCGACAGGTGCTTCTCGATCTGCA  TCCTGGGATCAAAGCCATAGTGAAGGACAGTGATGGACAGCCGACGGCAGTTGGGATTCG  TGAATTGCTGCCCTCTGGTTATGTGTGGGAGGGCTAA | Red: Cherry  Yellow: FasL  Blue: GS Linker  Green: PreS1 48  Purple: 2A  Black: Blast  Orange-7NNK |
| 217-3-GFP | ATGGGGCAGAATCTTTCCACCAGCAATCCTCTGGGATTCTTTCCCGACCACCAGTTGGAT  CCATTGTGGACGAGTAATAAGAAGTCGTCTGGATCAGGCGGTGGCGGTTCAGGAGGTGGT  GGCTCAGGCGGAGGAGGTTCCGGTGGCGGCGGCAGTGGTGGTGGAGGCTCTGGTGGTGGA  GGCTCTGGAGGCGGAGGTTCAGGAGGTGGTGGATCTGGAGGAGGTGGATCTGTGAGCAAG  GGCGAGGAGCTGTTCACCGGGGTGGTGCCCATCCTGGTCGAGCTGGACGGCGACGTAAAC  GGCCACAAGTTCAGCGTGTCCGGCGAGGGCGAGGGCGATGCCACCTACGGCAAGCTGACC  CTGAAGTTCATCTGCACCACCGGCAAGCTGCCCGTGCCCTGGCCCACCCTCGTGACCACC  CTGACCTACGGCGTGCAGTGCTTCAGCCGCTACCCCGACCACATGAAGCAGCACGACTTC  TTCAAGTCCGCCATGCCCGAAGGCTACGTCCAGGAGCGCACCATCTTCTTCAAGGACGAC  GGCAACTACAAGACCCGCGCCGAGGTGAAGTTCGAGGGCGACACCCTGGTGAACCGCATC  GAGCTGAAGGGCATCGACTTCAAGGAGGACGGCAACATCCTGGGGCACAAGCTGGAGTAC  AACTACAACAGCCACAACGTCTATATCATGGCCGACAAGCAGAAGAACGGCATCAAGGTG  AACTTCAAGATCCGCCACAACATCGAGGACGGCAGCGTGCAGCTCGCCGACCACTACCAG  CAGAACACCCCCATCGGCGACGGCCCCGTGCTGCTGCCCGACAACCACTACCTGAGCACC  CAGTCCGCCCTGAGCAAAGACCCCAACGAGAAGCGCGATCACATGGTCCTGCTGGAGTTC  GTGACCGCCGCCGGGATCACTCTCGGCATGGACGAGCTGTACAAGTAA | Grey: 217-3  Blue: GS Linker  Green: GFP |
| 217-4-GFP | ATGGGGCAGAATCTTTCCACCAGCAATCCTCTGGGATTCTTTCCCGACCACCAGTTGGAT  CCATTGCGGCGGGTGGCGGAGTTTTCGTCTGGATCAGGCGGTGGCGGTTCAGGAGGTGGT  GGCTCAGGCGGAGGAGGTTCCGGTGGCGGCGGCAGTGGTGGTGGAGGCTCTGGTGGTGGA  GGCTCTGGAGGCGGAGGTTCAGGAGGTGGTGGATCTGGAGGAGGTGGATCTGTGAGCAAG  GGCGAGGAGCTGTTCACCGGGGTGGTGCCCATCCTGGTCGAGCTGGACGGCGACGTAAAC  GGCCACAAGTTCAGCGTGTCCGGCGAGGGCGAGGGCGATGCCACCTACGGCAAGCTGACC  CTGAAGTTCATCTGCACCACCGGCAAGCTGCCCGTGCCCTGGCCCACCCTCGTGACCACC  CTGACCTACGGCGTGCAGTGCTTCAGCCGCTACCCCGACCACATGAAGCAGCACGACTTC  TTCAAGTCCGCCATGCCCGAAGGCTACGTCCAGGAGCGCACCATCTTCTTCAAGGACGAC  GGCAACTACAAGACCCGCGCCGAGGTGAAGTTCGAGGGCGACACCCTGGTGAACCGCATC  GAGCTGAAGGGCATCGACTTCAAGGAGGACGGCAACATCCTGGGGCACAAGCTGGAGTAC  AACTACAACAGCCACAACGTCTATATCATGGCCGACAAGCAGAAGAACGGCATCAAGGTG  AACTTCAAGATCCGCCACAACATCGAGGACGGCAGCGTGCAGCTCGCCGACCACTACCAG  CAGAACACCCCCATCGGCGACGGCCCCGTGCTGCTGCCCGACAACCACTACCTGAGCACC  CAGTCCGCCCTGAGCAAAGACCCCAACGAGAAGCGCGATCACATGGTCCTGCTGGAGTTC  GTGACCGCCGCCGGGATCACTCTCGGCATGGACGAGCTGTACAAGTAA | Grey: 217-4  Blue: GS Linker  Green: GFP |

**Supplementary Figure 1**


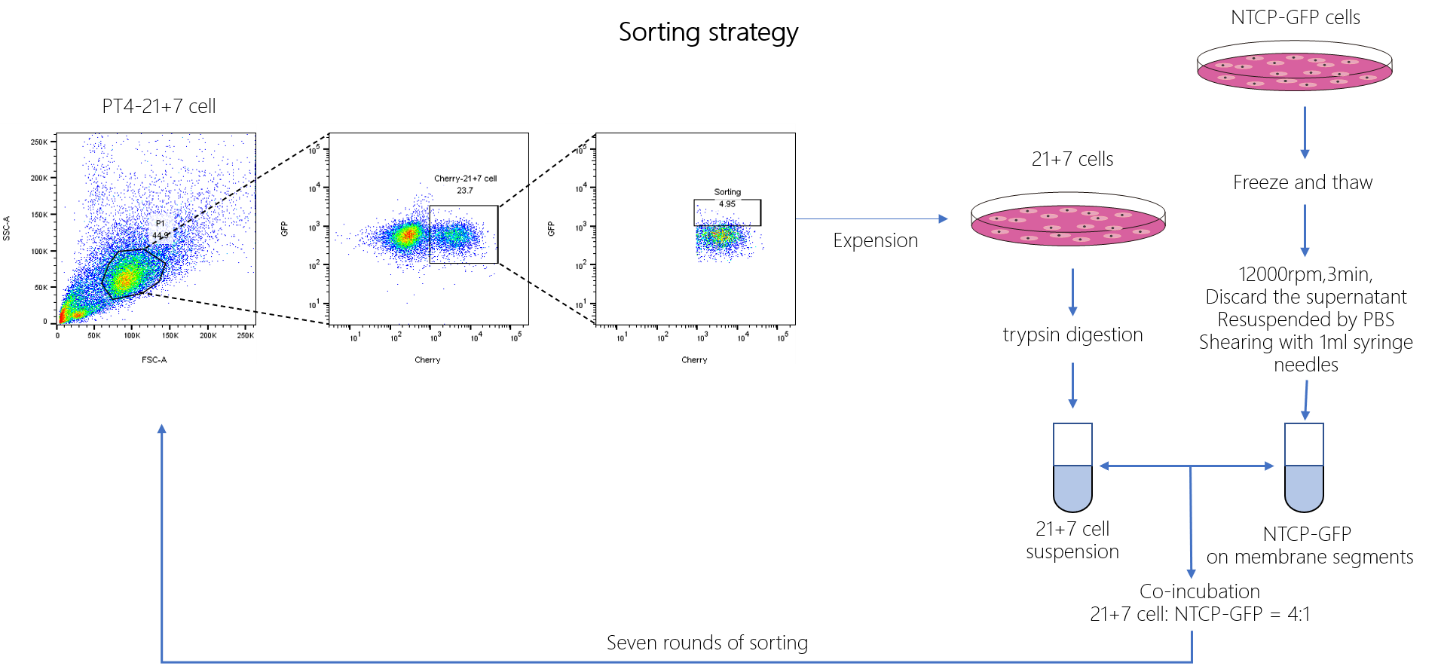


**Supplementary Fig. 1. The sorting strategy of the library.** PT4-21+7 cells were incubated with the membrane segments carrying NTCP-GFP and sorted by FACS. A sorting gate was set so that 5% of the red-fluorescence cells with the highest green fluorescence could be selected.

**Supplementary Figure 2**


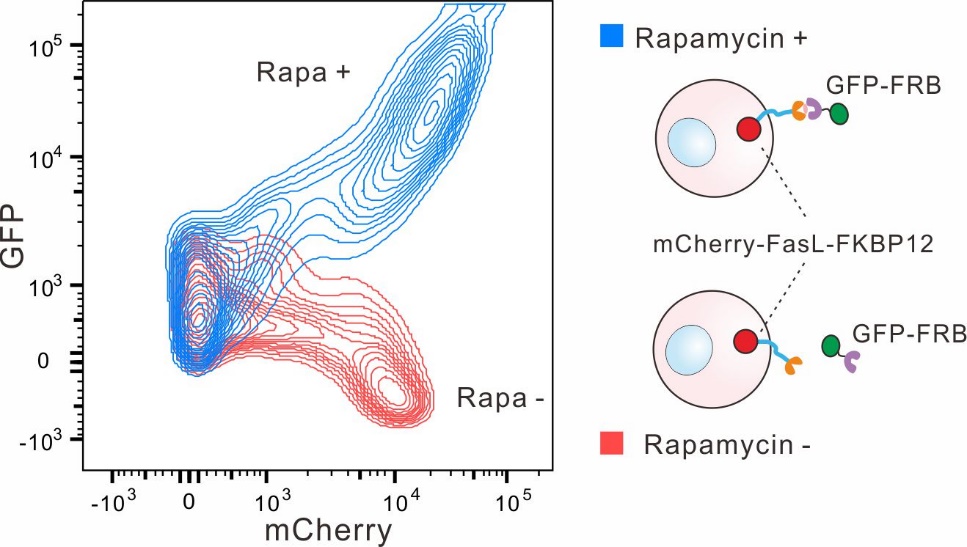


**Supplementary Fig. 2. Detection of FRB-FKPB12 interaction by the cell-surface display.** In the absence of rapamycin, there was no interaction between FRB and FKBP12, and the cells could only emit red fluorescence. When rapamycin is present, green fluorescence can be detected on the cells.

**Supplementary Figure 3**


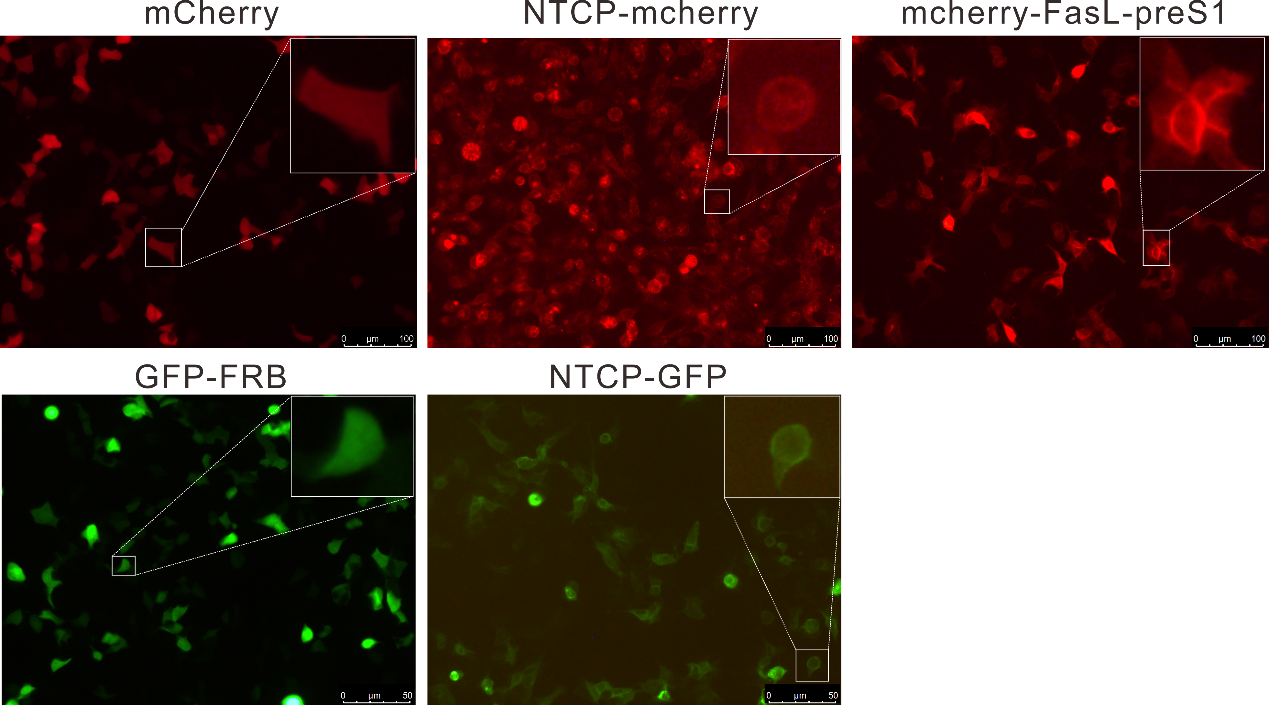


**Supplementary Fig. 3. NTCP and the preS1 peptide can be displayed on the cell surface.** NTCP-Cherry, NTCP-GFP, and Cherry-FasL-preS1 distribute along the membranes, while mCherry and GFP-FRB are distributed equally in the whole cells.

**Supplementary Figure 4**

**
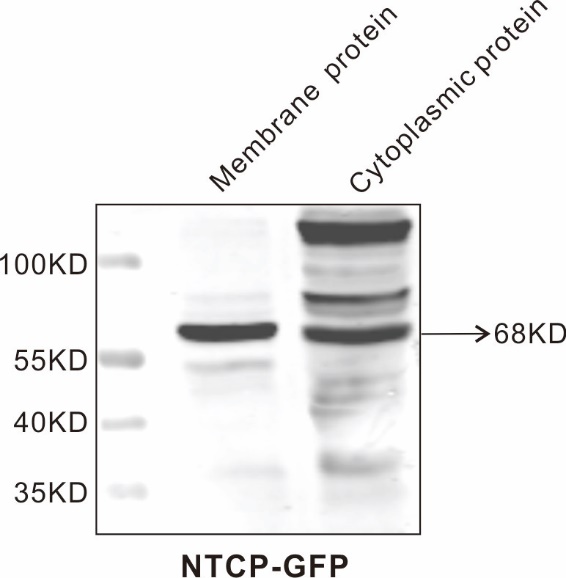
**

**Supplementary Fig. 4. Detection of NTCP-GFP in cells.** Membrane protein and cytoplasmic protein of 293-NTCP-GFP cells were extracted by using the Membrane and Cytosol Protein Extraction Kit (Beyotime, China), respectivley. The extracted proteins were detected by Western blot using an NTCP antibody.

**Supplementary Figure 5**


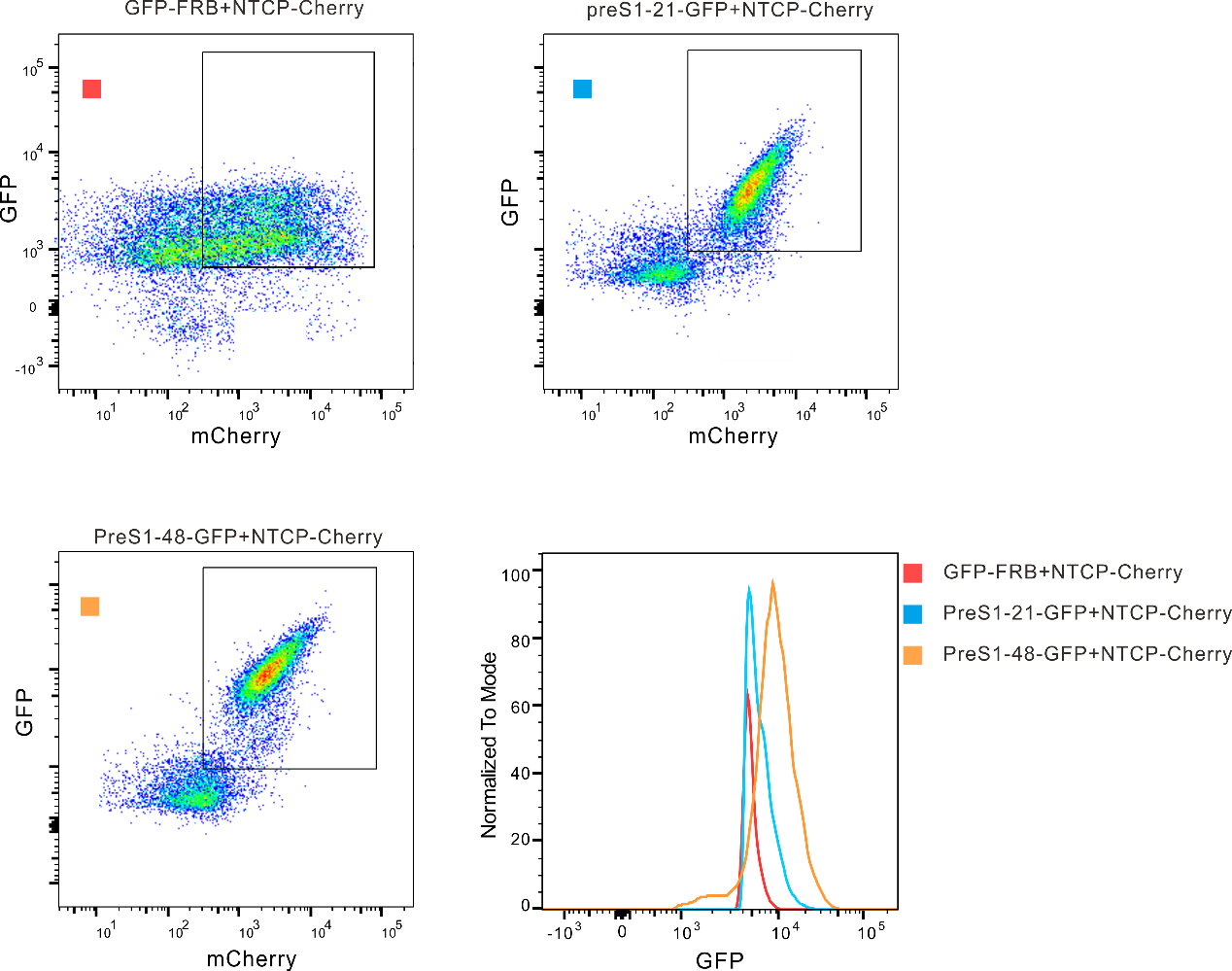


**Supplementary Figure 5. The validation assay can distinguish peptides with different affinity for NTCP.** NTCP-Cherry was displayed on the HEK293 cell surface, and GFP-fused peptides were used as bait proteins. NTCP-Cherry cells do not bind the GFP-FRB and bind more preS1-48-GFP than preS1-21-GFP.

**
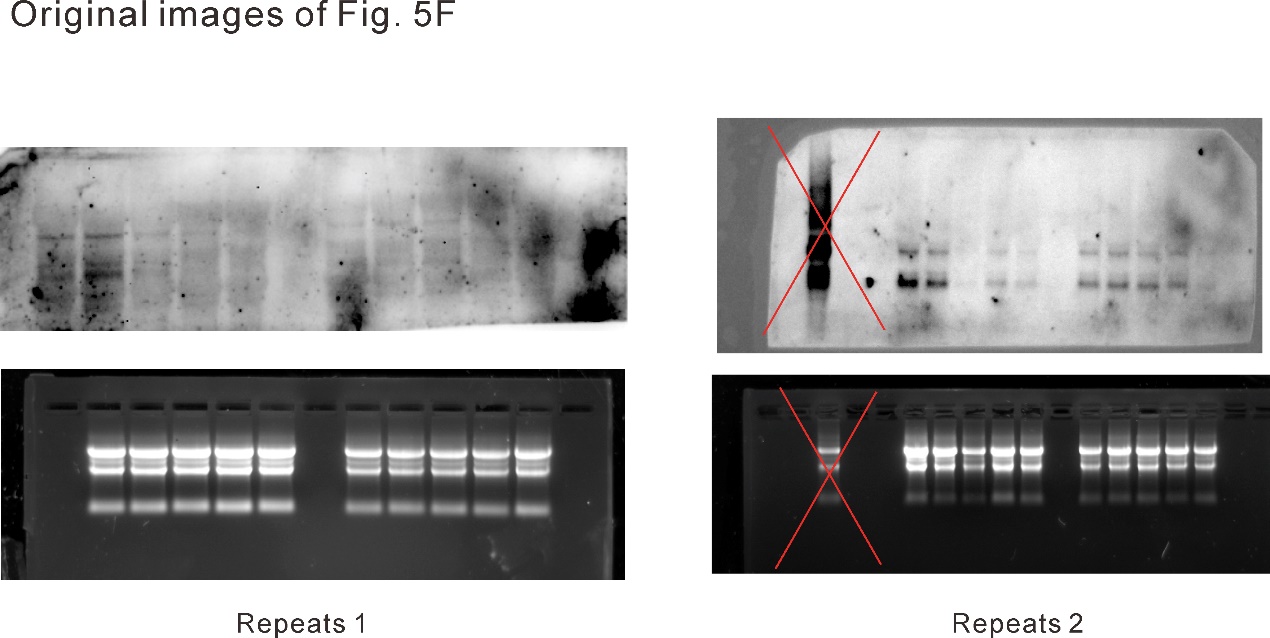
**
